# Easy and effective test to evaluate tear-film stability for self-diagnosis of dry eye syndrome: blinking tolerance time (BTT)

**DOI:** 10.1186/s12886-020-01686-5

**Published:** 2020-11-04

**Authors:** Hyung Bin Hwang, Yong Ho Ku, Eun Chul Kim, Hyun Seung Kim, Man Soo Kim, Ho Sik Hwang

**Affiliations:** 1 Department of Ophthalmology, Incheon St. Mary’s Hospital, College of Medicine, The Catholic University of Korea, Incheon, Republic of Korea; 2 Department of Ophthalmology, Chuncheon Sacred Heart Hospital, College of Medicine, Hallym University, Chuncheon, Republic of Korea; 3 Department of Ophthalmology, Bucheon St. Mary’s Hospital, College of Medicine, The Catholic University of Korea, Bucheon, Republic of Korea; 4 Department of Ophthalmology, Seoul St. Mary’s Hospital, College of Medicine, The Catholic University of Korea, Seoul, Republic of Korea; 5 Department of Ophthalmology, Eunpyeong St. Mary’s Hospital, College of Medicine, The Catholic University of Korea, Seoul, Republic of Korea; 6 Department of Ophthalmology, Yeouido St. Mary’s Hospital, College of Medicine, The Catholic University of Korea, Seoul, Republic of Korea

**Keywords:** Tear breakup time, Blinking tolerance time, Tear film instability, Diagnostic tool of dry eye

## Abstract

**Background:**

The tear film breakup time (tBUT) is a clinical evaluation of evaporative dry eye disease assessed by instilling topical fluorescein into the eyes. In the present study, we introduce a new diagnostic test, blinking tolerance time (BTT), for self-evaluation of tear-film stability. We compared the results with the tBUT and validated the BTT test for self-assessment of tear film instability.

**Methods:**

This was a prospective controlled study involving 212 eyes of 106 participants 20–79 years of age. A total of 114 eyes of 57 dry eye patients and 98 eyes of 49 healthy subjects were included in the study. All patients and subjects were administered the following tests to diagnose dry eye disease: Ocular Surface Disease Index, BTT, tBUT, slit-lamp examination, corneal stain score, and Schirmer I test (without anesthesia). Patients and subjects were instructed not to blink for as long possible after reset blinking. The time interval between the reset blink and the next blink was measured. The mean of 3 tBUT values in both the right and left eyes was defined as tBUT_BE_. Correlations between the BTT and tBUT_BE_ were also evaluated. To evaluate the diagnostic efficacy of the BTT and tBUT tests, receiver operating characteristic (ROC) curves were generated to obtain a cutoff score, and the sensitivities of the tests against the specificity at all possible thresholds were plotted.

**Results:**

Spearman’s correlation analysis revealed a significant weakly positive correlation between BTT and tBUT_BE_ (*r* = 0.447; *p* = 0.000). The intraclass correlation coefficient (ICC) of the tBUT was 0.679 (95% confidence interval [CI]: 0.575–0.765) and the ICC of the BTT was 0.904 (95% CI: 0.867–0.932). The area under the ROC curve did not significantly differ between the tBUT_BE_ (0.678) and BTT (0.628, *p* = 0.641). When the cutoff value of the BTT test was set to 8.1 s, the sensitivity was 63.3% and the specificity was 56.1%.

**Conclusion:**

The BTT test is a simple, inexpensive, and effective method for self-diagnosing dry eye that can also be used in the clinical setting.

## Background

Tear film instability plays a crucial role in the pathophysiology of dry eye disease. Thinning of the aqueous layer of the tear film due to reduced tear secretion from the lacrimal glands or thinning of the lipid layer of the tear film due to reduced meibum (oil) secretion from the meibomian glands leads to rapid evaporation and tear film breakup [1]. The tear film breakup time (tBUT) is traditionally measured to assess tear-film stability in the clinic. In the tBUT test, fluorescein dye is applied to the conjunctival sac and the time to tear breakup is evaluated while the patient eye is open under the cobalt blue light of a slit-lamp [2]. Although this method is effective, there are some disadvantages. First, the use fluorescein dye does not allow for observation of the physiological state of the ocular surface; moreover, the breakup time is dependent on the amount of fluorescein dye used, so that the tBUT value may differ accordingly. Second, it is sometimes difficult to determine when the tear film begins to breakup, so the tBUT test has low reproducibility [3]. Finally, although the probability is very low, the use of fluorescein dye poses a risk of infection.

There have been some attempts to overcome these limitations of the tBUT test. Recently, a noninvasive tBUT test was developed to measure the tear breakup time without fluorescein dye using the Keratograph 5 M instrument (K5M; Oculus Optikgerate, Wetzlar, Germany) [4] or DR-1α (Kowa, Nagoya, Japan). While this method allows for tBUT to be performed noninvasively, these instruments may not be readily available.

A staring contest is a well-known game in which 2 people face each other and the person who can delay blinking for the longest time wins the game. We considered that this game could be an inexpensive and simple method to assess tear-film stability. The aim of the present study was to introduce a new test, the blinking tolerance time (BTT) test, that could be used for self-assessment of tear-film stability. To evaluate the clinical usefulness of the BTT test, the results were compared with tBUT measurements obtained with the traditional method using fluorescein dye.

## Methods

### Selection of participants

This study followed the tenets of the Declaration of Helsinki, and the Institutional Review Board of Chuncheon Sacred Hospital and Incheon St. Mary’s Hospital approved the protocol. Patients were recruited at the Cornea Service of Chuncheon Sacred Hospital. The study included healthy subjects (*n* = 49) and dry eye patients (*n* = 57) matched for age and sex. For the dry eye group, the inclusion criteria were age ≥ 20 years and at least mild dry eye symptoms (an Ocular Surface Disease Index [OSDI] score ≥ 13) and low tear film break-up time (TBUT < 5 s), or low Schirmer I score (< 10 mm per 5 min without anesthesia), or corneal punctate fluorescein staining (Oxford staining score of > 1) in at least 1 eye. Exclusion criteria were a history of ocular injury, infection, non-dry eye ocular inflammation, trauma, surgery within the prior 6 months, or the presence of uncontrolled systemic disease. For healthy subjects, inclusion criteria were no dry eye syndrome (no typical dry eye syndrome symptoms, tBUT > 5 s, Schirmer I score > 10 mm, Oxford staining score < 1), and the exclusion criteria were the same.

### Evaluation of dry eye

The examinations were performed as follows: OSDI, BTT, slit-lamp examination, tBUT, corneal stain, and Schirmer I (without anesthesia) test. Using the OSDI score, subjective symptoms of dry eye were graded numerically from 0 to 4, and the sum of these scores was used in the analyses [5]. The OSDI, developed by the Outcomes Research Group at Allergan (Irvine, CA, USA), is a 12-item questionnaire yielding information on vision-related functioning [5]. Each item is scored on a 5-point scale, resulting in a total OSDI score ranging from 0 (*no symptoms*) to 100 (*maximal symptoms*). Slit-lamp examination was performed to identify exclusion criteria. For the tBUT test, a sterile fluorescein strip (Haag, Heidelberg, Germany) moistened with balanced salt solution was applied to the inferior fornix of both eyes. After approximately 2 min, the subjects were asked to blink several times to ensure mixing of the dye and tear fluid, and the time interval between the last blink and the appearance of the first dry spot of tear film was recorded. To evaluate intra-examiner repeatability, 3 consecutive measurements were obtained by the same clinician. The tBUT was recorded to the nearest 0.1 s using a digital stopwatch. The mean of three tBUT measurements was determined for each eye. The average of the mean value for both eyes was defined as tBUT_BE_. The lowest mean tBUT value between the 2 eyes was defined as tBUTs. Corneal fluorescein staining was also performed under cobalt blue slit-lamp illumination. The Oxford grading scheme [6] was applied to evaluate the ocular surface damage. Approximately 15 min after the tBUT test, the Schirmer test was performed without topical anesthesia.

### Blinking tolerance time test

The BTT test was performed in eyes of all participants by the same clinician. The BTT test was conducted in a silent and windless examination room. The temperature and humidity of the room were carefully controlled (23 °C ~ 26 °C and 40% ~ 60%, respectively). Air conditioners and electric fans were not in use during the examination. For the BTT tests, subjects and patients sat in the examination chair with their eyes closed. Immediately after opening both eyes, the investigator instructed each participant to fix his or her eyes to a visual target 3 m away and keep their eyes open until they had to blink due to ocular pain. The investigator measured the time during which the eyes were open without blinking to within 0.1 s using a stopwatch, i.e., the time interval between the last blink and the unavoidable next blink. The unavoidable next blink resulted from the participant experiencing irritation or pain. The participants were instructed not to over-open or unnaturally open their eyes, and the BTT test was performed with the eyes open as naturally as possible. To measure the intra-examiner repeatability, the BTT test results were calculated based on 3 consecutive measurements obtained by the same clinician. Between each measurement, the participants were given a break for at least 30 s to ensure stable and reproducible data. If reflex tearing occurred during a BTT test, the results were excluded from the analysis.

### Order of the diagnostic testing procedures

The order of the testing procedures during the ophthalmic examinations was as follows:

1. Subjective interview regarding symptoms of dry eye (i.e., OSDI) and recording of patient medical history.
2. BTT test, repeated 3 times.
3. Slit-lamp evaluation of the cornea, conjunctiva, eyelids, and Meibomian glands.
4. Schirmer I test (without anesthesia).
5. tBUT test using fluorescein dye, repeated 3 times.
6. Fluorescein staining of the cornea.

### Statistical analysis

All statistical analyses were performed using SAS software (ver. 9.0; SAS Institute, Cary, NC, USA). A value of *P <* 0.05 was considered statistically significant, and 95% confidence intervals (CIs) were calculated. The mean ± standard deviation (SD) of age, OSDI, Schirmer values (mm), tBUT_BE_, and tBUT_S_ values were calculated for all participants. For all participants, we also calculated the mean ± SD of the difference between the BTT and tBUT_BE_, and of the difference between the BTT and tBUT_S_. The distribution of the BTT values was measured, and the Kolmogorov-Smirnov test was performed to confirm that the data were normally distributed. The Wilcoxon signed-rank test was performed to determine whether there was a statistically significant difference between the mean values of the BTT and tBUT_BE_ measured in all participants and between the mean values of the BTT and tBUT_S_. Spearman’s correlation test was performed to confirm the correlation between the BTT and tBUT_BE_, and Spearman’s rho values were calculated. In addition, Spearman’s correlation was used to determine whether there was a correlation between the BTT and OSDI values.

The mean values for age, sex, OSDI, Schirmer values, corneal staining score (according to the Oxford grading scheme), tBUT_BE_, tBUT_S_, and BTT were calculated for the control group and dry eye patient groups. Also, the difference between the BTT and tBUT_BE_, and between the BTT and tBUT_S_, were calculated for both groups, and the mean value of these differences was calculated. The Mann-Whitney U test was then used to confirm whether the mean values were significantly different between the 2 groups. In addition, the Wilcoxon signed-rank test was used to determine whether there was a statistically significant difference between the BTT and tBUT_BE_ in each group.

For repeated measurements of the tBUT and BTT, intraclass correlation coefficients (ICCs) were calculated to determine the consistency between the measurements (i.e., the repeatability of the measurements within the same participant). The ICCs of the BTT and tBUT were used to determine the intra-examiner repeatability (ICC ≥ 0.75 indicated good reliability). To evaluate and maximize the diagnostic efficacy of the BTT and tBUT tests, receiver operating characteristic (ROC) curves were generated to obtain a cutoff score, and the sensitivities of the tests against the specificity at all possible thresholds were plotted. The area under the ROC curve (AUC) was used to determine how the tests performed as diagnostic instruments. An AUC value of 0.5 indicated a completely ineffective diagnostic test, and a value of 1.0 indicated a maximally effective diagnostic test [5, 7].

## Results

Table 1 shows the age, sex, OSDI, Schirmer test, corneal staining, tBUT_BE_, BUT_S_, BTT, BTT-tBUT_BE_, and BTT-tBUT_S_ data for the normal and dry eye patient groups.

**Table 1:** Basic characteristics of enrolled subjects

	Total	Healthy subjects	Dry eye syndrome patients	*p*-value
Number of subjects or patients	106	49	57	
Number of eyes	212	98	114	
Age (years)	52.8 ± 13.5	51.9 ± 13.2	53.6 ± 13.7	0.200^*^
Sex (M:F)	40:66	21:28	19:38	0.159†
Ocular Surface Disease Index (OSDI)	43.6 ± 23.6	38.5 ± 24.9	47.8 ± 21.8	0.029^*^
Schirmer (mm)	10.1 ± 6.2	11.6 ± 6.5	8.8 ± 4.4	0.002^*^
Cornea stain (Oxford)	0.8 ± 0.8	0.3 ± 0.6	1.2 ± 0.8	0.000^*^
tBUT_BE_ (sec)	12.2 ± 9.2	14.5 ± 9.9	10.3 ± 8.2	0.000^*^
tBUTs (sec)	8.8 ± 5.3	10.2 ± 5.7	7.5 ± 4.7	0.003^*^
BTT (sec)	13.5 ± 17.1	18.1 ± 22.8	9.6 ± 8.1	0.024^*^
BTT-tBUT_BE_ (sec)	1.3 ± 17.0	3.6 ± 22.9	−0.7 ± 9.1	0.534^*^
BTT- tBUTs (sec)	4.8 ± 16.8	7.9 ± 23.3	2.1 ± 6.8	0.626^*^

^*^Mann-Whitney U test

†Fisher’s exact test

*tBUT*_*BE*_ mean tear break-up time of right eye and left eye, *BUT*_*S*_ shorter tear break-up time of right eye and left eye, *BTT* Blinking tolerance time

The mean values of tBUT_BE_ and BTT for all 106 participants were 12.2 ± 9.2 s and 13.5 ± 17.1 s, respectively. There was no statistically significant difference between the 2 mean values (*p* = 0.215; Wilcoxon signed-rank test). The mean value of TBUT_S_ was 8.8 ± 5.3 s, which was significantly shorter than that of BTT (*p* = 0.003; Wilcoxon signed-rank test). The mean value of the difference between the tBUT_S_ and BTT in each participant was 4.8 ± 16.8 s.

**Fig. 1:**
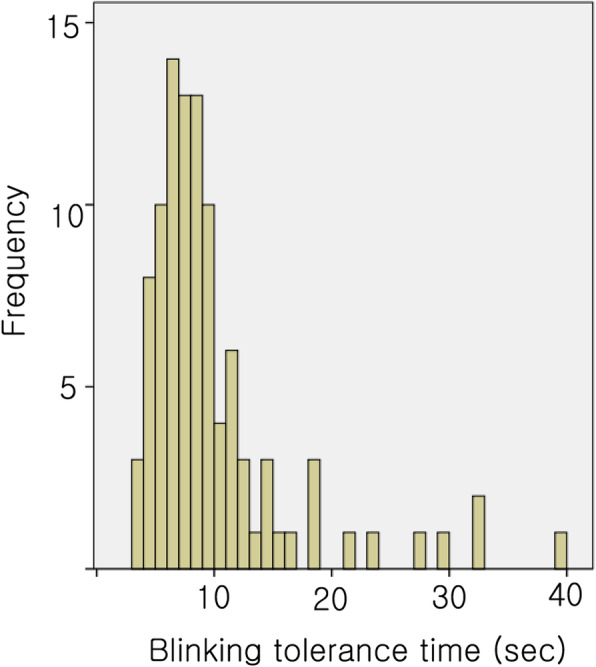
The blinking tolerance time (BTT) values exhibited a left-skewed distribution. The Kolmogorov-Smirnov test indicated that the BTT test values were not normally distributed (*p* = 0.000)

**Fig. 2:**
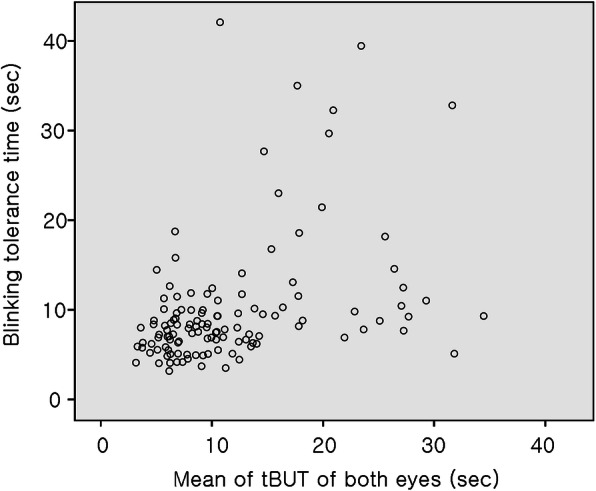
The BTT and tear breakup time (tBUT) without anesthesia showed a statistically significant positive correlation (*r* = 0.447; *p* = 0.000; Spearman’s correlation analysis)

Figure 1 shows that the distribution of BTT values was skewed to the left, and the BTT values were not normally distributed according to the results of the Kolmogorov-Smirnov normality test (*p* = 0.000). Figure 2 shows the correlation between the BTT and tBUT_BE_ values. Spearman’s correlation analysis showed a statistically significant, weakly positive correlation between the BTT and tBUT_BE_ values (*r* = 0.447; *p* = 0.000). There was no statistically significant correlation between the BTT and OSDI values among all participants (*r* = − 0.160; *p* = 0.105; Spearman’s correlation test). The ICC of the tBUT values was 0.679 (95% CI: 0.575–0.765) and the ICC of the BTT values was 0.904 (95% CI: 0.867–0.932).

The age and sex distributions did not differ significantly between the normal and dry eye groups (*p* = 0.200 and 0.159, respectively; Mann-Whitney U test and Fisher’s exact test) (Table 1). The OSDI values and corneal stain were higher in the dry eye group compared with the normal group. The Schirmer, tBUT_BE_, and tBUTs values were lower in the dry eye group. The BTT was significantly longer in the normal group than in the dry eye group (18.1 ± 22.8 s vs 9.6 ± 8.1 s, *p* = 0.024, Mann-Whitney U test).

In the normal subject group, the BTT and tBUT_BE_ did not differ significantly (*p* = 0.483, Wilcoxon signed-rank test). In the dry eye group, the BTT and tBUT_BE_ values also did not differ significantly (*p* = 0.324, Wilcoxon signed-rank test). Further, BTT-tBUT_BE_ did not differ significantly between the normal subjects and dry eye group (*p* = 0.534, Wilcoxon signed-rank test). The BTT-tBUT_S_ did not differ significantly between the normal subjects and dry eye group (*p* = 0.626, Wilcoxon signed-rank test).

**Fig. 3:**
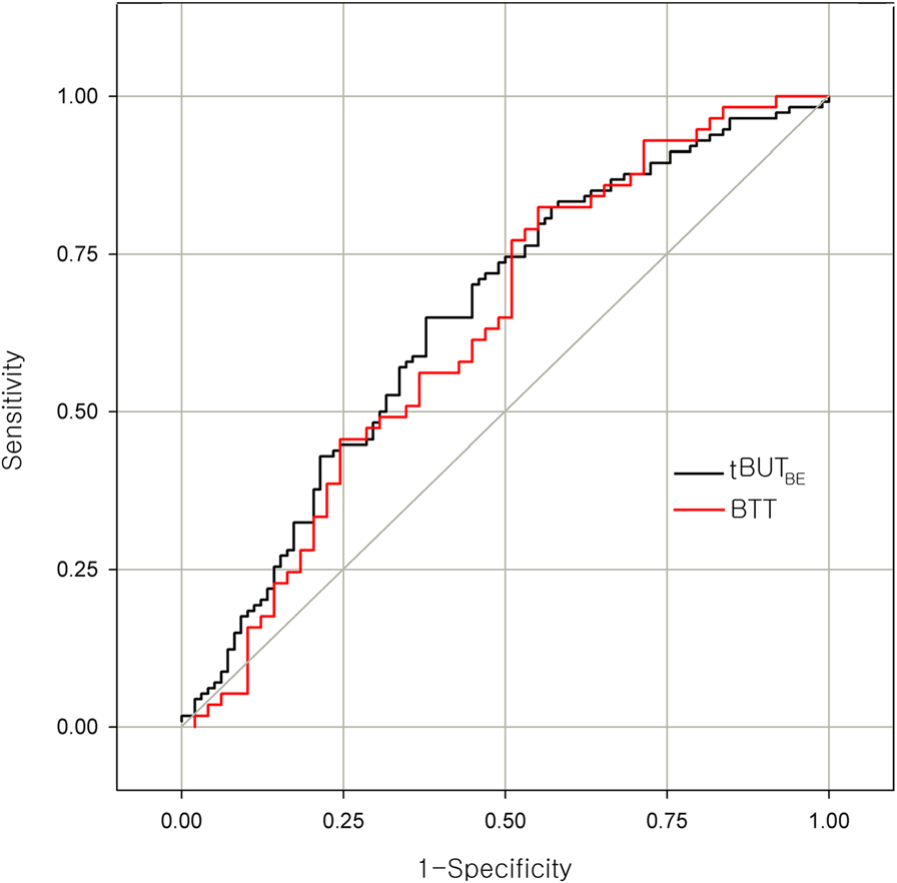
Receiver operating characteristic (ROC) curve of tear breakup time without anesthesia (TBUT_[BE]_) and the BTT. The area under the ROC curve (AUC) of the tBUT_BE_ was 0.678 and the AUC of the BTT was 0.628. The difference between these 2 AUC values did not reach statistical significance (*p* = 0.641)

The ROC curves of the tBUT_BE_ and BTT values are shown in Fig. 3. The AUC of the tBUT_BE_ was 0.678 and that of the BTT was 0.628, with no statistically significant difference between these values (*p* = 0.641; method of DeLong et al. [8]). When the cutoff value of BTT was set to 8.1 s, the sensitivity of the BTT test was 63.3% and the specificity was 56.1%.

## Discussion

We report a new diagnostic test, called the BTT test, for evaluating tear film instability. This test evaluates the stability of the tear film indirectly by measuring the time interval between eye blinks when the participant has been asked to refrain from blinking for as long as possible before feeling a foreign body sensation or irritation. Tear film instability is a key mechanism of dry eye disease [9], and new, effective diagnostic approaches that are inexpensive and noninvasive would be beneficial.

The present study revealed no statistically significant difference between the tBUT_BE_ and BTT among all participants, and a significant positive correlation between the 2 measurements. Further, there was no significant difference in the AUC between the tBUT_BE_ and BTT values. The BTT values for the dry eye group were significantly shorter than those for the normal control group. Thus, the BTT test can be used for self-diagnosis of dry eye disease.

Sophisticated scientific diagnostic instruments, such as the TearLab system (TearLab, San Diego, CA, USA) and LipiView (TearScience, Morrisville, NC, USA) interferometry systems, which incorporate remarkable technologic innovations, have been developed for clinical use. Dry eye disease is a prevalent disorder that represents a major public health burden due to its negative impact on patient vision and quality of life. The BTT test is a simple method for self-diagnosis of this disease with high reliability. This inexpensive new diagnostic tool can also be used widely by clinicians to diagnose dry eye disease.

Several studies have been conducted to determine the association between eye blinking and dry eye disease. Pult et al. [10] performed a computerized analysis of video recordings of spontaneous blinking in normal and dry eye patients, and reported that the inter-blinking time (IBT) differed significantly between the 2 groups (4.0 ± 2.0 s for the normal group and 1.5 ± 0.9 s for the dry eye group). The IBT in their study corresponds to the BTT used in our study. The BTT differs from the IBT, however, in that the participant is asked not to blink after the reset blink until he or she feels ocular pain. In addition, our study focused on analyzing the correlation between the BTT and tBUT tests without using complex equipment, such as video capture and analysis equipment, in contrast to the study by Pult et al. [10]. We first confirmed that there was no significant difference between the BTT and tBUT_BE_ values; second, we found a significant positive correlation between the 2 tests; third, the ICC of the BTT test was superior to that of the tBUT_BE_ test; and finally, there was no significant difference between the AUCs of the 2 tests. These findings confirmed that the BTT test is appropriate for dry eye diagnosis, having comparable utility to the tBUT test. The diversity of the blink rate is related to the severity of dry eye disease. Previous studies reported that the blink rate increased in the aqueous-deficient type of dry eye disease; furthermore, the increased blink rate was positively correlated with corneal staining and subjective dry eye symptoms, and negatively correlated with the tBUT [11, 12]. Several reports have described the mechanism underlying blinking in patients with dry eye disease.

Ocular surface temperature decreases over time after blinking [13]. The tear film begins to destabilize after blinking due to evaporation, which cools the ocular surface [14]. The rate of evaporation is increased in patients with dry eye due to the instability of the lipid layer [15]. The ocular surface temperature may therefore be decreased in patients with dry eye due to tear film instability immediately after blinking compared with normal controls [16]. Stimulation of cold thermoreceptors in the cornea between blinks can result in basal tear secretion, and such stimulation in normal controls is related to ocular comfort and wetness [17]. Reduced sensitivity of these thermoreceptors in patients with dry eye, who already have a cooler-than-normal corneal temperature of 34 °C, can decrease the physiologic tearing stimulation, thus leading to increased subjective dry eye symptoms such as pain and a stinging sensation [12].

The concept of the BTT is different from the blinking interval in a relaxed state. The BTT test measures the time until the participant blinks after sensing a foreign body sensation or irritation. The BTT measurement is therefore consistent with the concept of tear film stability, which is not related to the pain threshold or nociceptor activation. It is possible that the BTT is related to the pain threshold and nociceptor activation, and that this test is thus less objective than the tBUT test, being relatively strongly associated with the symptoms afflicting patients with dry eye. Therefore, we analyzed the correlation between the BTT and OSDI results, but detected no significant association.

In the present study, we aimed to demonstrate the efficacy of the BTT test, a new method for diagnosing dry eye, compared with the conventional tBUT test. The BTT test can serve as a useful dry eye diagnostic tool as an adjunct to the tBUT test: first, the mean values of the BTT and tBUT in the normal control group did not differ significantly from each other; second, there was no statistically significant difference in the AUC between the BTT and tBUT; and third, both the tBUT and the BTT were significantly shorter in patients with dry eye than in the normal controls. The BTT and tBUT measurements, however, showed a weakly positive correlation, for the following reasons: first, both the BTT and tBUT tests may not be highly reproducible; second, the pain threshold may differ among participants; and third, an understanding of and willingness to undergo the BTT test (which involves blinking naturally after feeling a foreign body sensation or irritation) may also differ among individuals.

The BTT test has several advantages compared with the tBUT test. First, it can be used as a self-test of dry eye disease. The BTT test is very easy and simple to perform, such that anyone can use it to evaluate dry eye disease without assistance from a clinician. In this study, we found no significant difference between the BTT and tBUT results among all participants, and the BTT was shorter in patients with dry eye than in normal subjects. Because the AUC of the BTT was 0.628, it was insufficient to diagnose dry eye disease alone, but could be used as a self-test for screening purposes. Second, the BTT test was superior to the tBUT test with respect to the reproducibility of the results. In our study, the ICC of the BTT test was 0.904 and that of the tBUT test was 0.679; the BTT test was therefore more reproducible than the tBUT test. Third, the BTT test better reflects the physiologic status of the tear film, because no fluorescein dye is needed for the testing procedure.

The BTT test has some weaknesses. First, the BTT is affected not only by tear film stability, but also by each individual pain threshold; a person with a high pain threshold will have a longer BTT and vice versa. As such, we expected that the OSDI, which reflects the subjective symptoms of patients with dry eye, would be significantly correlated with the BTT, but instead, the results of BTT test showed a significant positive correlation with the results tBUT test. Second, the BTT test examines both eyes simultaneously, so that the condition of each eye cannot be measured separately, unlike in the tBUT or Schirmer tests (both of which test 1 eye at a time). Of course, we can measure the BTT in 1 eye at a time, with the other eye closed, but this method would not reflect the physiologic status of the tear film.

## Conclusions

The tBUT_BE_ and BTT values among all participants did not differ significantly, and a significant positive correlation was detected between the 2 measurements. In addition, the ICC of the BTT test was superior to that of the tBUT test, and the AUC of the BTT test was not significantly different from that of the TBUT test. Most importantly, the BTT values were significantly shorter in dry eye patients than in normal subjects. These results suggest that the BTT test can be used as an adjunct to existing tests for evaluating dry eye disease as well as for self-diagnosis of the disorder.

## Data Availability

The datasets used and analyzed during the current study are available from the corresponding author on reasonable request.

## Abbreviations

OSDI: Ocular Surface Disease Index
BTT: Blinking tolerance time
tBUT: Tar break-up time
ICC: Intraclass correlation coefficient
CIs: Confidence intervals
SD: Standard deviation
ROC: Receiver operating characteristic
AUC: Area under the curve
IBT: Inter-blinking time

## References

[1] Bron AJ, de Paiva CS, Chauhan SK, Bonini S, Gabison EE, Jain S (2017). TFOS DEWS II pathophysiology report. Ocul Surf.

[2] Norn MS (1969). Desiccation of the precorneal film. I. Corneal wetting-time. Acta Ophthalmol.

[3] Nichols KK, Mitchell GL, Zadnik K (2004). The repeatability of clinical measurements of dry eye. Cornea..

[4] Markoulli M, Duong TB, Lin M, Papas E (2018). Imaging the tear film: a comparison between the subjective Keeler Tearscope-plus and the objective oculus(R) Keratograph 5M and LipiView(R) interferometer. Curr Eye Res.

[5] Schiffman RM, Christianson MD, Jacobsen G, Hirsch JD, Reis BL (2000). Reliability and validity of the ocular surface disease index. Arch Ophthalmol.

[6] Bron AJ, Evans VE, Smith JA (2003). Grading of corneal and conjunctival staining in the context of other dry eye tests. Cornea..

[7] Simpson TL, Situ P, Jones LW, Fonn D (2008). Dry eye symptoms assessed by four questionnaires. Optom Vis Sci.

[8] DeLong ER, DeLong DM, Clarke-Pearson DL (1988). Comparing the areas under two or more correlated receiver operating characteristic curves: a nonparametric approach. Biometrics..

[9] Herrero-Vanrell R, Peral A (2007). International dry eye workshop (DEWS). Update of the disease. Arch Soc Esp Oftalmol.

[10] Pult H, Riede-Pult BH, Murphy PJ (2013). The relation between blinking and conjunctival folds and dry eye symptoms. Optom Vis Sci.

[11] Gumus K, Crockett CH, Pflugfelder SC (2010). Anterior segment optical coherence tomography: a diagnostic instrument for conjunctivochalasis. Am J Ophthalmol.

[12] Rahman EZ, Lam PK, Chu CK, Moore Q, Pflugfelder SC. Corneal sensitivity in tear dysfunction and its correlation with clinical parameters and blink rate. Am J Ophthalmol 2015;160(5):858–866 e5. doi: 10.1016/j.ajo.2015.08.005. PubMed PMID: 26255576; PubMed Central PMCID: PMC4661092. 10.1016/j.ajo.2015.08.005 PMC4661092 26255576

[13] Efron N, Young G, Brennan NA (1989). Ocular surface temperature. Curr Eye Res.

[14] Craig JP, Singh I, Tomlinson A, Morgan PB, Efron N (2000). The role of tear physiology in ocular surface temperature. Eye..

[15] Mathers WD, Binarao G, Petroll M (1993). Ocular water evaporation and the dry eye. A new measuring device. Cornea..

[16] Purslow C, Wolffsohn J (2007). The relation between physical properties of the anterior eye and ocular surface temperature. Optom Vis Sci.

[17] Belmonte C, Gallar J (2011). Cold thermoreceptors, unexpected players in tear production and ocular dryness sensations. Invest Ophthalmol Vis Sci.

